# Supplementary material for: ssDNA accessibility of Rad51 is regulated by orchestrating multiple RPA dynamics
Source: Nat Commun. 2023 Jun 30;14:3864. doi: 10.1038/s41467-023-39579-y (PMC10313831; doi:10.1038/s41467-023-39579-y)
Supplement: Supplementary file 1 — Supplementary Information [file 41467_2023_39579_MOESM1_ESM.pdf]

Supplementary Information

ssDNA accessibility of Rad51 is regulated by orchestrating multiple RPA dynamics

Jiawei Ding[1,†], Xiangting Li[2,†], Jiangchuan Shen[3,†], Yiling Zhao[1,5], Shuchen Zhong[4], Luhua Lai[1,4], Hengyao Niu[3,*], and Zhi Qi[1,*]

[†]Equal contribution

[1]Center for Quantitative Biology, Peking-Tsinghua Center for Life Sciences, Academy for Advanced Interdisciplinary Studies, Peking University, Beijing 100871, China

[2]Department of Biomathematics, University of California, Los Angeles, California 90023, USA

[3]Department of Molecular and Cellular Biochemistry, Indiana University, Bloomington, IN 47401, USA

[4]BNLMS, College of Chemistry and Molecular Engineering, Peking University, Beijing 100871, China

[5]Present address: Institute of Systems Biomedicine, School of Basic Medical Sciences, Peking University Health Science Center, Beijing 100191, China

*To whom correspondence should be addressed: zhiqi7@pku.edu.cn; hniu@indiana.edu

Table of contents

1. Supplementary Methods
   1.1 RPA dynamic binding to long ssDNA can be simulated by a generalized random sequential adsorption (RSA) model
2. Supplementary Figures
3. Supplementary References

1. Supplementary Methods

1.1 RPA dynamic binding to long ssDNA can be simulated by a generalized random sequential adsorption (RSA) model

To elucidate the biophysical mechanism of RPA dynamic binding to long ssDNA, we began by establishing a continuous-time discrete Markov chain model (Fig. 4a-b). Random sequential adsorption (RSA) models were initially proposed by Paul Flory [1] to describe irreversible adsorption processes of large molecules to a liquid-solid interface. If the interface is chosen to be one-dimensional (1D) and a finite length, the so-called 1D RSA model will provide an ideal basis for further modelling the processes of protein binding to DNA. Because this model precisely captures a key property, in which each nucleotide (nt) of ssDNA cannot be occupied by more than two protein molecules. This property differentiates DNA-relevant reactions from elementary chemical reactions described by the mass action law. And more importantly, it leads to incomplete occupation even if the proteins are oversaturated. Relevant theoretical consequences of applying RSA-like model to DNA-protein reaction kinetics are also known as the McGhee-von Hippel model [2-4]. This type of model has been applied to study similar biophysics settings [5], where a standard mean field approximation is employed to obtain accurate parameters of the kinetic parameters. In order to reveal finer structures such as the gap distribution, we implemented an exact stochastic sampling approach to this model.

In our work of RPA, we adapted the model according to our current knowledge of RPA binding modes to obtain full elements simulation results in which multiple binding modes and volume exclusion effects are all taken into consideration. Based on the previous references [6-8], we assumed that RPA has two binding modes, 20-nt mode and 30-nt mode, representing partial binding mode (PBM) and full-length binding

mode (FLBM) (Fig. 4a). RPA initially binds to a 20-nt ssDNA with a rate of k_1 (unit s^{-1}, the concentration of DNA and RPA incorporated), and dissociates to ssDNA with a rate of k_{-1} (unit s^{-1}). This is the 20-nt mode. k_1 is assumed to be constant in the model because ssDNA Curtains maintain a laminar flow of RPA, which allows the concentration of free RPA to be constant. One possible scenario for the 20-nt mode is the DBD-A, DBD-B, and DBD-C together binding to ssDNA [7]. The DBD-D subsequent binding leads to the 30-nt mode (Fig. 4a). When an RPA molecule is in the 20-nt mode, DBD-D starts to bind to an extra 10-nt ssDNA with a rate of k_2 (unit s^{-1}), and dissociates to ssDNA with a rate of k_{-2} (unit s^{-1}). This is the 30-nt mode (FLBM). The recent reference [7,9] suggested that the 30-nt mode is related to two different conformations, which are the extended conformation or the U-shape conformation (Fig. 4a). Here, we need to highlight the polarity of RPA binding to ssDNA. RPA always aligns along DNA in the same direction [7].

Due to the existence of multiple binding modes, RPA molecules exhibit interesting kinetic features such as facilitated exchange and desorption. With the advancement of computational power, we can now use the novel dynamic language Julia [10] to carry out exact stochastic simulations that allow for all the possible behaviors of RPA-ssDNA interactions that are known to us. The relevant codes are available through link (https://github.com/hsianktin/RPA_model).

In this model (Fig. 4b), the state of ssDNA fragment is represented by a vector of length L, taking values in $0,1$, where 0 represents that this nt is not occupied and 1 represents the nt being occupied. Each RPA must take up $l = 20$ nts for initial binding with DNA. And if the local state of DNA permits, it can further occupy another $\Delta l = 10$ nts in the 3' direction. We assume that each consecutive unoccupied segment of

length l recruits one RPA at the rate of k_1 (unit s^{-1}, the concentration of RPA incorporated), and that the total binding rate of RPA

$$v_1 = k_1 \sum_{j=1}^{L-l+1} 1_{0^{\times l}}(\text{state}[j, j+1, \dots, j+l-1]) .$$

The summation $\sum_{j=1}^{L-l+1} 1_{0^{\times l}}(\text{state}[j, j+1, \dots, j+l-1])$ standing for the number of consecutive unoccupied segments of length l on the ssDNA. For further occupying another 10 nts, we assign a rate parameter k_2 and calculate the overall rate by

$$v_2 = k_2 \sum_{q_j} 1_{0^{\times \Delta l}}\left(\text{state}\left[q_j + l, q_j + l + 1, \dots, q_j + l + \Delta l - 1\right]\right),$$

where q_j represents the leftmost position of the jth bound RPA in the 20-nt mode.

In terms of the unbinding pathway, we assume that the 30-nt mode must be reopened into the 20-nt mode before detaching from DNA, and the rate is assumed to be k_{-2}. And the desorption rate for the 20-nt mode is denoted as k_{-1}. The overall rates v_{-2} and v_{-1} are calculated according to the following formula: let p_j iterate over all 30-nt mode RPAs and let q_j iterate over all 20-nt mode RPAs as before,

$$v_{-2} = k_{-2} \sum_{p_j} 1,$$

$$v_{-1} = k_{-1} \sum_{q_j} 1.$$

For each moment, the total possible reaction rate v_{tot} (s^{-1}) is:

$$v_{tot} = v_1 + v_{-1} + v_2 + v_{-2} \tag{1}$$

Summation is taken over all possible reactions under the specific configuration. All the underlying reactions occur stochastically according to their exponentially distributed waiting times whose distribution parameter being corresponding reaction

rates. In other words, the waiting time between two adjacent reactions T (s) follows an exponential distribution, which is a probability density function (pdf):

$$\mathrm{pdf}(T = t) = v_{tot} e^{-v_{tot} t} \quad (2)$$

And the mean value of T (s) is:

$$\langle T \rangle = \frac{1}{v_{tot}} \quad (3)$$

After each reaction happens, the state of DNA is changed, and the number of possible reactions should be evaluated again based on the current state of DNA. Gillespie algorithm [11] is applied to the system for sampling trajectories from this stochastic model.

In experiments, the contour length of each ssDNA is unknown and varied. Simulation samples the ratios of DNA covered by 20-nt mode RPA (c_{20}) and 30-nt mode RPA (c_{30}) at given time points. The ratio of naked DNA is then $c_n = 1 - c_{20} - c_{30}$. In order to compare simulation outcomes with experimental data, we introduce additional parameters α and β, such that the relative extension of ssDNA is calculated as:

$$x_r(t) = \alpha \times c_{30}(t) + \beta \times c_{20}(t) + 1 \times c_n(t)$$

Then, we scale the extension of ssDNA at the 30-min time point to unit 1.

$$x(t) = \frac{x_r(t)}{x_r(30\mathrm{min})}$$

Now, $x(t)$ is comparable with the experimental results. We compared the weighted squared differences between the mean extension-time curves of experimental data and simulation outcomes. The parameters used for simulation are determined through sampling a wide range of plausible parameters. The parameter with the least squared error is chosen (Supplementary Fig. 5).

Due to its significant computational cost, we adopted the following optimization strategy. We optimize the $\log_{10}$ of corresponding parameters. Within the experimental range of 40-min, and L = 5,000-nt, kinetic parameters should be at least 1×10^{-6} s^{-1}. The total length L of DNA is set to be 5,000 nts to reduce computational costs while maintaining good approximation to realistic scenarios.

We adopted a discrete gradient descent method to optimize the parameters. Let $k_{i,n}$ denote the current estimate of kinetic parameter k_i. We obtain 50 samples from each possible combinations of kinetic parameters $(k_1, k_{-1}, k_2, k_{-2})$ from the collection $\{10^{-0.5}k_1, 10^0 k_1, 10^{0.5}k_1\} \times \cdots \times \{10^{-0.5}k_{-2}, 10^0 k_{-2}, 10^{0.5}k_{-2}\}$. The next step is determined by the combination that minimizes the loss function with respect to experimental data. The range of non-kinetic parameters α and β is fixed, with α ranging from 0.5 to 10, and β ranging from 0.2 ~ 2 times of α. The loss function is chosen to be a weighted squared loss, where the weight is given by

$$w(t) = \begin{cases} 1, & 1200\,s < t \leq 2000\,s \\ 2, & 2000\,s < t \leq 2400\,s \end{cases}$$

The choice of this weight leverages the tractability of experimental data to obtain the intuitively best fitting of the experimental observations.

The loss function we chose does not admit a probabilistic interpretation. Therefore, there is no precise confidence interval defined for each parameter. We plot the minimum weighted loss associated with every type of parameter (Supplementary Fig. 5), to determine whether the minimum is reached or whether the range of parameters are appropriate. If the best parameter under current precision is determined, we optimize the parameter by refining the range of parameters and repeating the process.

This process ends when we find the simulation results being close enough to experimental curves (Fig. 4c).

2. Supplementary Figures

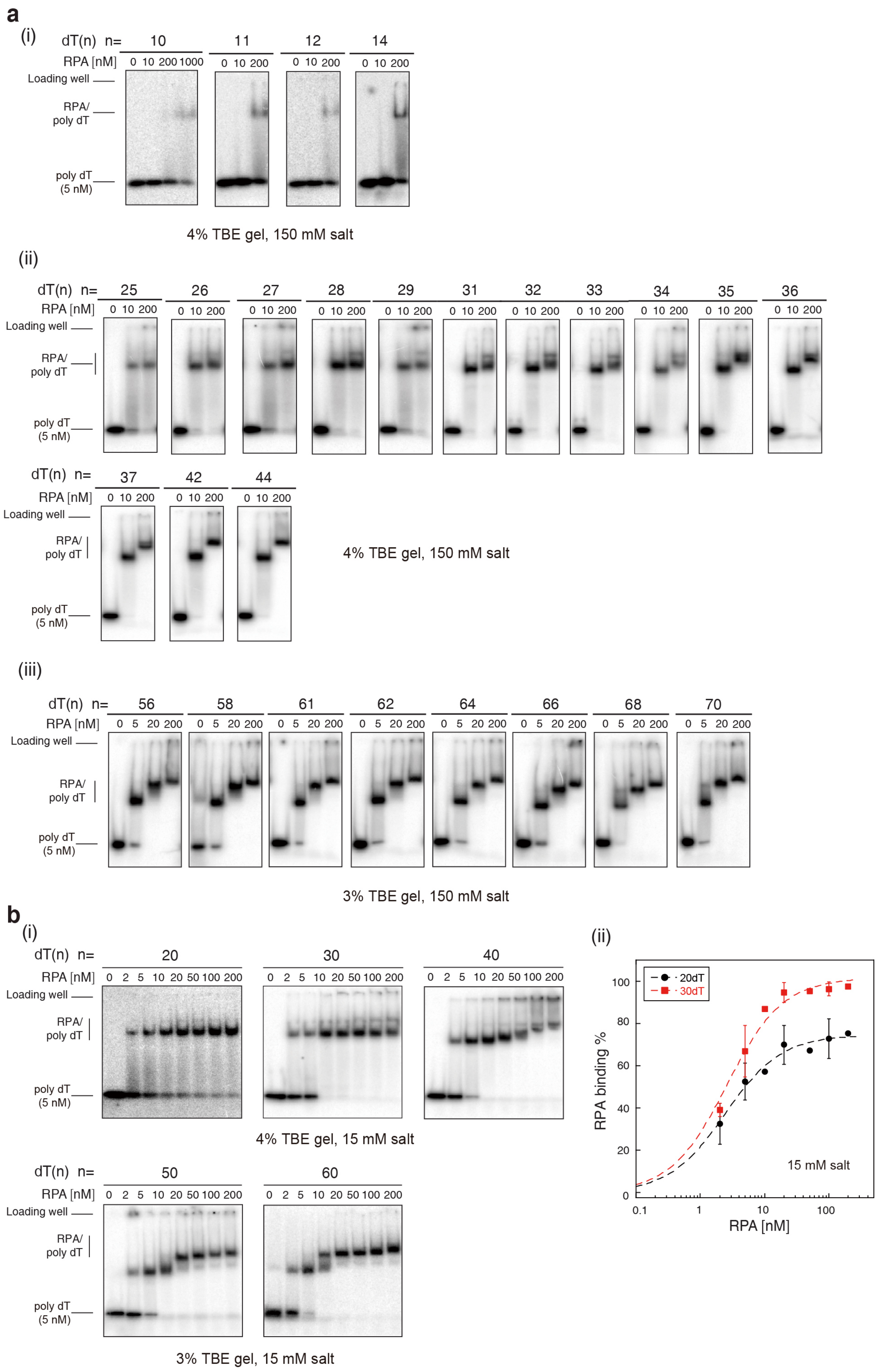

Supplementary Figure 1. EMSA assays of RPA binding to various poly-dT ssDNA substrates. (**a**) Titration of RPA (0-1000 nM) to 5 nM 5'-labeled $dT_{(10)}$ ssDNA and titration of RPA (0-200 nM) to 5 nM 5'-labeled $dT_{(n)}$ ssDNA, n = 11, 12, 14 (i); n = 25, 26, 27, 28, 29, 31, 32, 33, 34, 35, 36, 37, 42, 44 (ii); n = 56, 58, 61, 62, 64, 66, 68, 70 (iii), at 150 mM KCl. All the experiments were repeated three times. (**b**) Titration of RPA (0-200 nM) to 5 nM 5'-labeled $dT_{(n)}$ ssDNA at 15 mM KCl, n = 20, 30, 40, 50, 60 (i), and quantification of RPA binding percent to $dT_{(20)}$ and $dT_{(30)}$ (ii). Data are presented as mean ± SD in b(ii). *N* = 3 independent experiments for each experimental condition in b(ii). Source data are provided as a Source Data file.

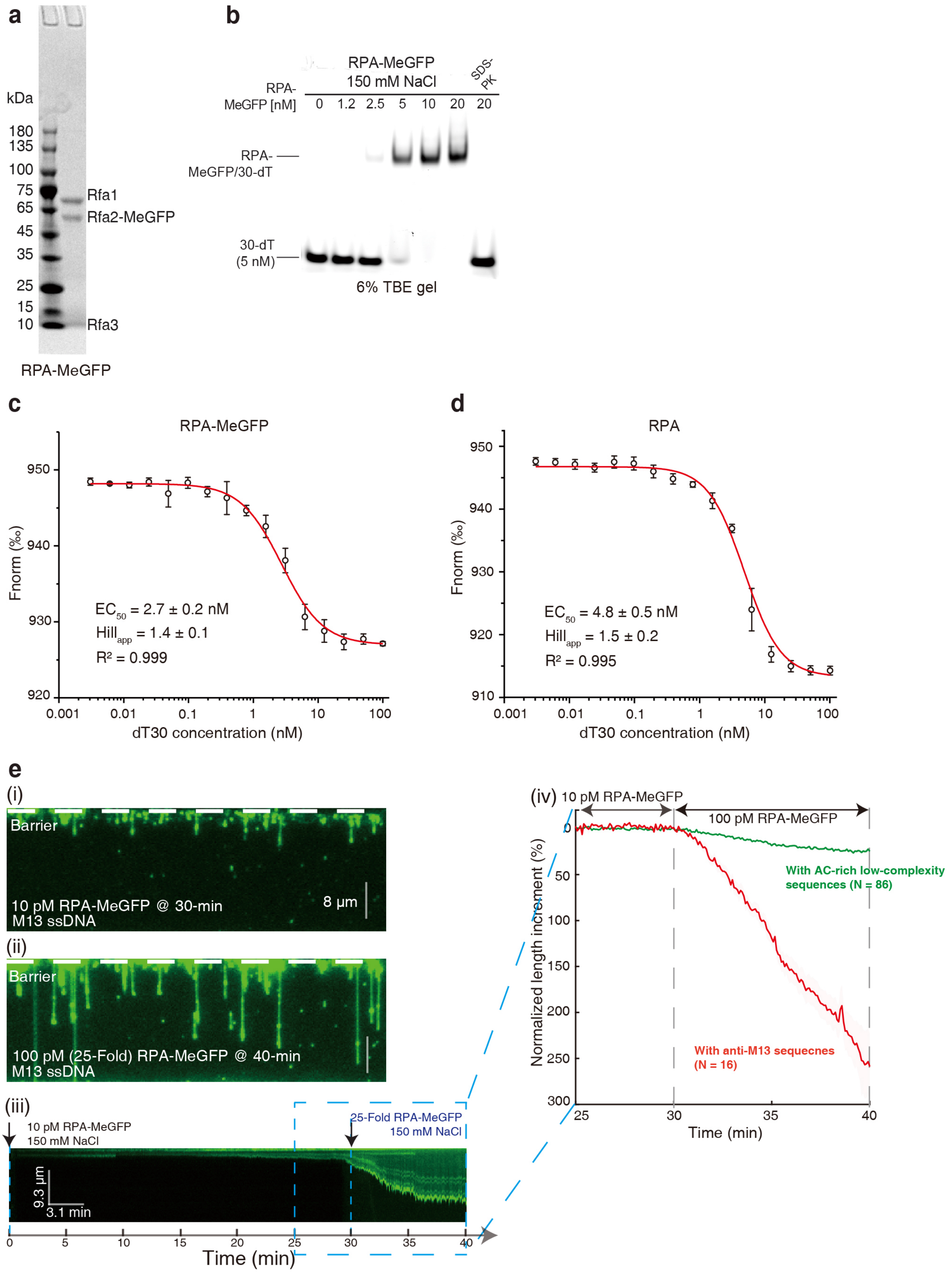

Supplementary Figure 2. RPA-MeGFP binding to ssDNA substrates with different sequences. (**a**) Purified RPA-MeGFP was analyzed on a 4-15% gradient SDS-PAGE. (**b**) Titration of RPA-MeGFP (0-20 nM) to 5nM 5'-labeled $dT_{(30)}$ ssDNA.

(**c-d**) Quantification of MST assays for the titration of $dT_{(30)}$ ssDNA to 50 nM RPA-MeGFP (**c**) and RPA (**d**). (**e**) Binding dynamics of RPA-MeGFP on M13 ssDNA. Wide-field TIRFM images of ssDNA Curtains with M13 ssDNA at 30-min time point with 0.4 mL/ flow of working buffer containing 10 pM RPA-MeGFP (i), or at 40-min time point with 10-fold RPA-MeGFP (100 pM) (ii). Representative TIRFM images of 10-fold RPA-MeGFP on M13 ssDNA at 150 mM NaCl were shown in (iii), and its length analysis (red) was in (iii), where length dynamic curve with 10-fold RPA-WT on AC-rich low-complexity ssDNA at 150 mM NaCl (green) from Fig. 3a were added for comparison. *N* represents the total trace number of ssDNA-RPA complexes end tracking examined over three times DNA Curtains experiments for each experimental condition in e. *N* = 3 independent experiments in c and d. Data are presented as mean ± SD in c and d, and mean ± SEM in e. Source data are provided as a Source Data file.

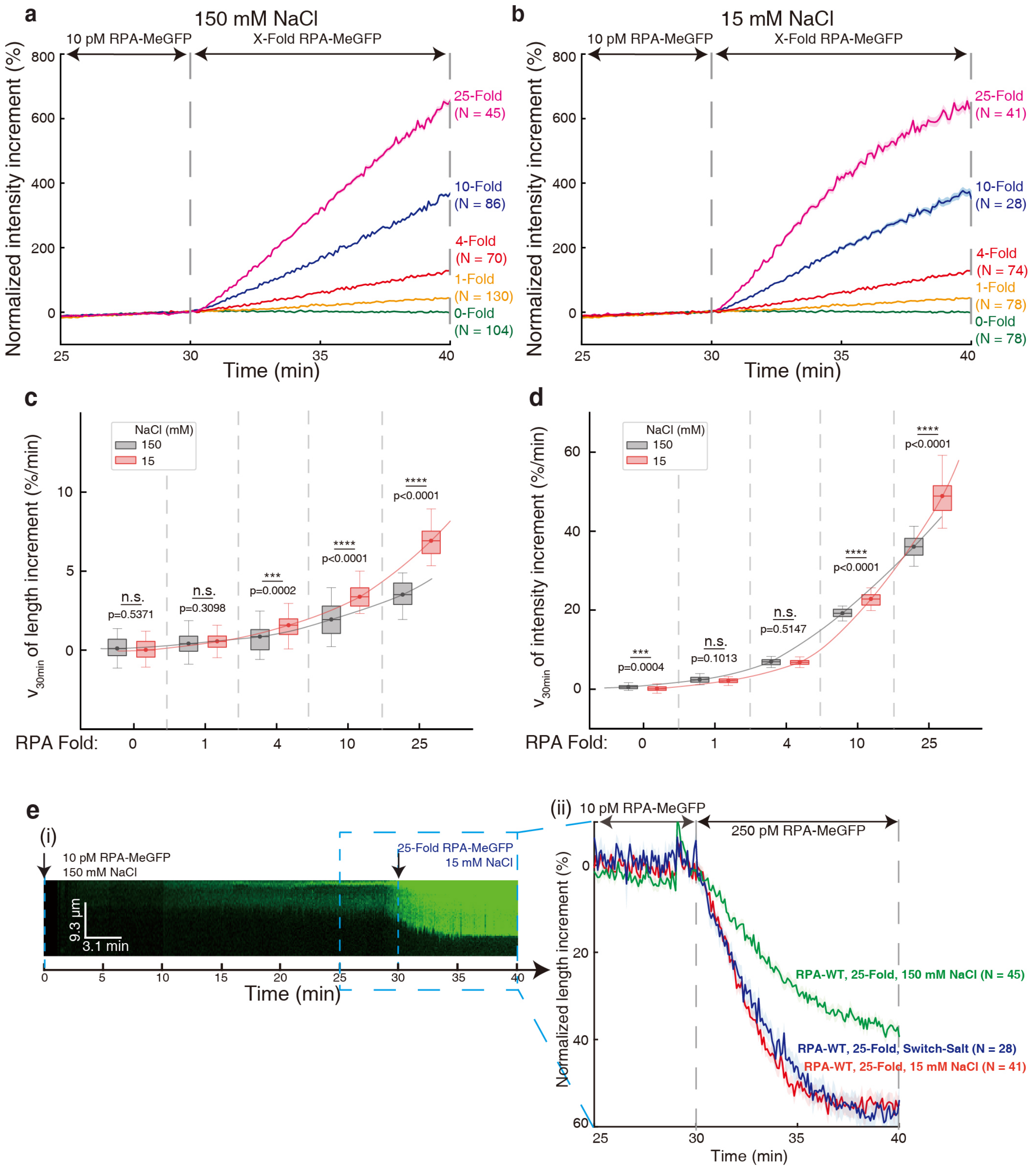

Supplementary Figure 3. Analysis of ssDNA Curtains results. (**a-b**) The intensity analysis of ssDNA-RPA complexes with 0, 1, 4, 10, 25-fold RPA from Fig. 3a-b. 150 mM NaCl in a and 15 mM NaCl in b. (**c-d**) Boxplot of initial velocity of extension length change in c or initial velocity of RPA-MeGFP intensity change in d. The initial velocity was defined by the rate of change within 30-min to 30.5-min. Exact *p*-value (from left to right): 0.5371, 0.3098, 0.0002, 3.8e-6, 4.3e-18, 0.0004, 0.1013, 0.5147, 1.6e-12,

1.2e-16. Boxplot style: middle line (mean), box range (0.25-0.75), whisker range (min-max), with outliers removed. Statistics: one-way ANOVA (analysis of variation). *P*-value style: GP: ⩾ 0.05 (ns), < 0.05 (*), < 0.01 (**), < 0.001 (***), < 0.0001 (****). (**e**) Binding dynamics of RPA-MeGFP with salt switch. Representative kymographs of salt switch experiment from 30-min 10 pM RPA-MeGFP at 150 mM NaCl to 10-fold RPA-MeGFP (100 pM) at 15 mM NaCl were shown in (i), and its length analysis (blue) was in (ii), where length dynamic curve with 10-fold RPA-WT at 150 mM NaCl (green) and 15 mM NaCl (red) condition from Fig. 3a-b were added for comparison. *N* for each condition in c-d was same with a-b. *N* represents the total trace number of ssDNA-RPA complexes end tracking examined over three times DNA Curtains experiments for each experimental condition in a, b, and e. Data are presented as mean ± SEM in a, b, and e. Source data are provided as a Source Data file.

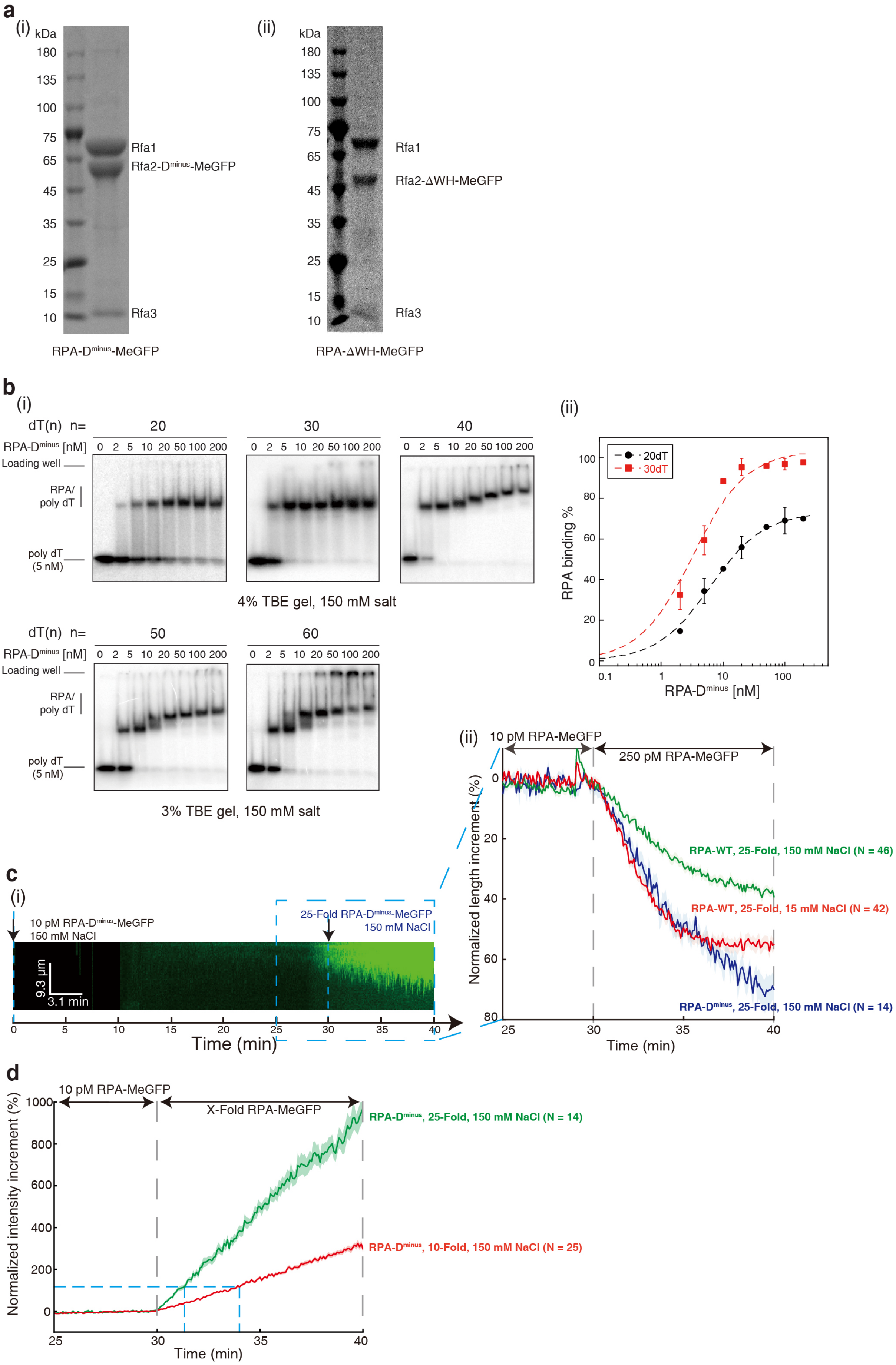
a
(i)
kDa
180
135
100
75
65
45
35
25
15
10
Rfa1
Rfa2-Dminus-MeGFP
Rfa3
RPA-Dminus-MeGFP
(ii)
kDa
180
135
100
75
65
45
35
25
15
10
Rfa1
Rfa2-ΔWH-MeGFP
Rfa3
RPA-ΔWH-MeGFP
b
(i)
dT(n) n= 20 30 40
RPA-Dminus [nM] 0 2 5 10 20 50 100 200
Loading well
RPA/ poly dT
poly dT (5 nM)
4% TBE gel, 150 mM salt
dT(n) n= 50 60
RPA-Dminus [nM] 0 2 5 10 20 50 100 200
Loading well
RPA/ poly dT
poly dT (5 nM)
3% TBE gel, 150 mM salt
(ii)
20dT
30dT
RPA binding %
RPA-Dminus [nM]
0.1
1
10
100
c
(i)
10 pM RPA-Dminus-MeGFP
150 mM NaCl
25-Fold RPA-Dminus-MeGFP
150 mM NaCl
9.3 μm
3.1 min
Time (min)
(ii)
10 pM RPA-MeGFP
250 pM RPA-MeGFP
Normalized length increment (%)
RPA-WT, 25-Fold, 150 mM NaCl (N = 46)
RPA-WT, 25-Fold, 15 mM NaCl (N = 42)
RPA-Dminus, 25-Fold, 150 mM NaCl (N = 14)
Time (min)
d
10 pM RPA-MeGFP
X-Fold RPA-MeGFP
Normalized intensity increment (%)
RPA-Dminus, 25-Fold, 150 mM NaCl (N = 14)
RPA-Dminus, 10-Fold, 150 mM NaCl (N = 25)
Time (min)

Supplementary Figure 4. Binding dynamics of RPA-D^{minus}. (**a**) Purified RPA-D^{minus}-MeGFP (i) and RPA-ΔWH-MeGFP (ii) was analyzed on a 4-15% gradient SDS-PAGE. (**b**) Titration of RPA-D^{minus} (0-200 nM) to 5 nM 5'-labeled dT$_{(n)}$ ssDNA at 150 mM KCl, n = 20, 30, 40, 50, 60 (i), and quantification of RPA binding percent to dT$_{(20)}$ and dT$_{(30)}$ (ii). (**c**) Binding dynamics of RPA-D^{minus} at 150 mM NaCl. Representative kymographs of 25-fold RPA-D^{minus} (250 pM) at 150 mM NaCl were shown in (i), and its length analysis (blue) was in (ii), where length dynamic curve with 25-fold RPA-WT at 150 mM NaCl (green) and 15 mM NaCl (red) condition from Fig. 3a-b were added for comparison. (**d**) Intensity analysis of RPA-D^{minus} at 150 mM NaCl. 10-fold in red and 25-fold in green. *N* represents the total trace number of ssDNA-RPA complexes end tracking examined over three times DNA Curtains experiments for each experimental condition in c and d. *N* = 3 independent experiments for each experimental condition in b(ii). Data are presented as mean ± SEM in c and d, and mean ± SD in b(ii). Source data are provided as a Source Data file.

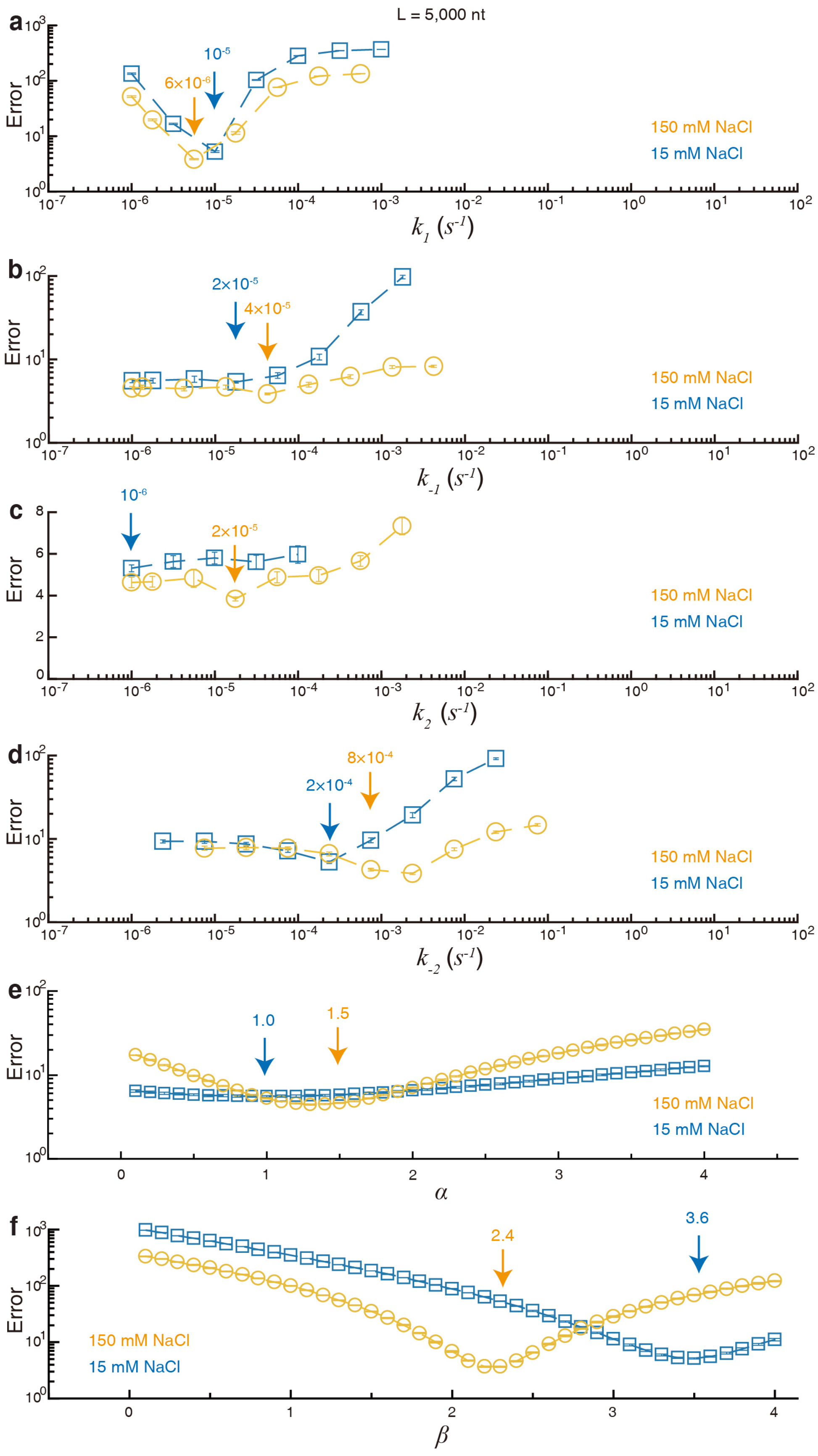

g

Simulation parameters		
NaCl [mM]	150	15
L (nt)	5,000	5,000
# of repeats	100	100
k_1 (s^{-1})	6×10^{-6}	10^{-5}
k_{-1}(s^{-1})	4×10^{-5}	2×10^{-5}
k_2 (s^{-1})	2×10^{-5}	10^{-6}
k_{-2}(s^{-1})	2×10^{-4}	8×10^{-4}
α	1.5	1.0
β	2.4	3.6

Supplementary Figure 5. Parameter determination of stochastic simulations. (**a-f**) The local loss function landscape with respect to single parameter. Here, we keep all the rest parameters invariant, and mutating the values of the focal parameter. For each given set of parameters, every 100 samples of trajectories are used to generate one sample of loss. We obtained the mean and standard error of the loss by 10 samples of the loss, summarized from 1,000 samples. We plot the mean and standard error of the resultant losses evaluated by the prescribed weighted mean square deviation as a function of the focal parameter. (**g**) Simulation parameters. Data are presented as mean ± SD in a-f. Source data are provided as a Source Data file.

a

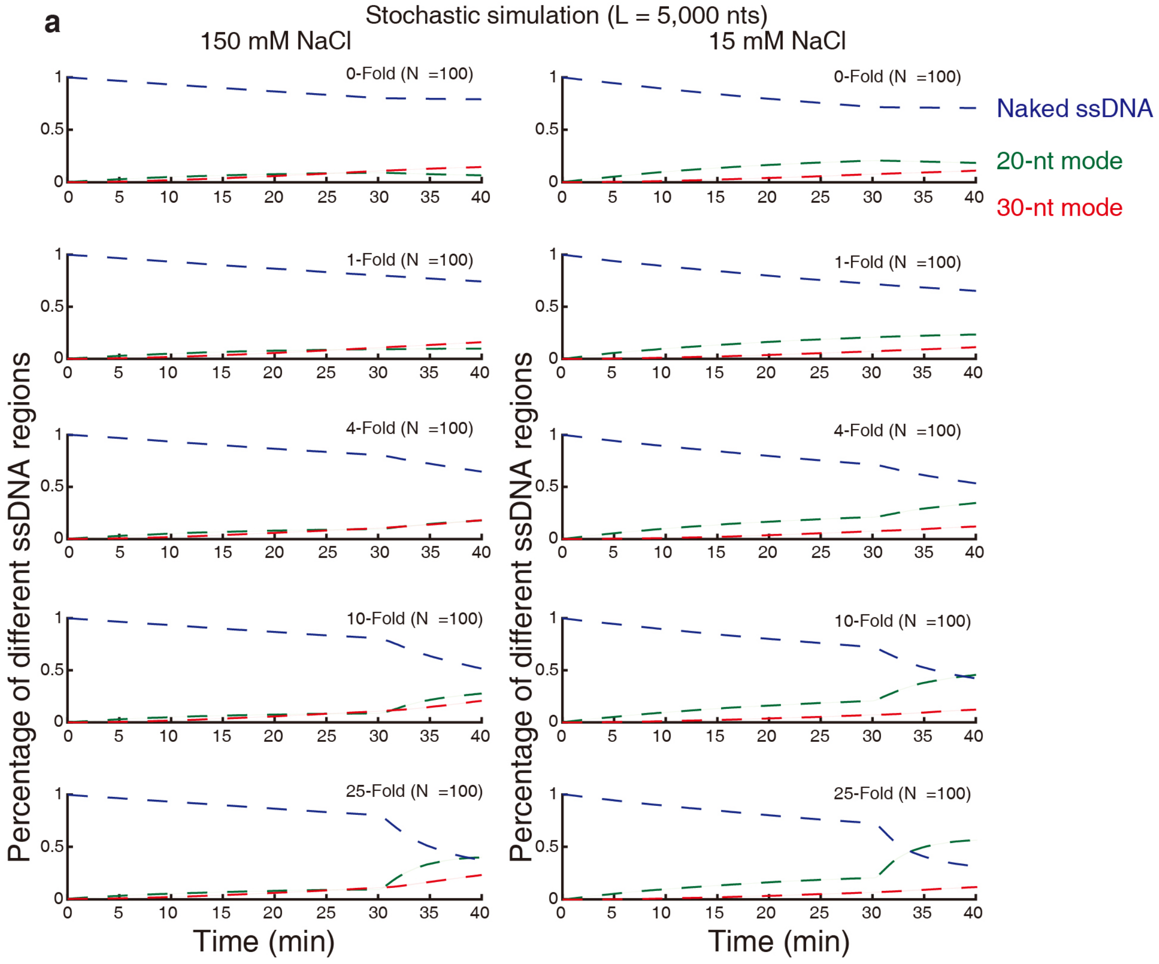

b @ 40-min

Length (nt)	5,000		5,000		5,000		5,000		5,000	
Fold	0		1		4		10		25	
NaCl [mM]	150	15	150	15	150	15	150	15	150	15
# $RPA_{30\text{-mode}}$	24	18	27	19	30	20	34	20	38	20
# $RPA_{20\text{-mode}}$	16	46	24	59	44	86	69	114	100	141
P_{20}	0.40	0.72	0.47	0.76	0.59	0.81	0.67	0.85	0.72	0.88
$\Delta N_{RPA,30\text{-}40min}$	0	0	10	13	33	42	64	71	98	98
Naked ssDNA (nt)	3,954	3,533	3,714	3,267	3,225	2,673	2,583	2,109	1,864	1,583

c

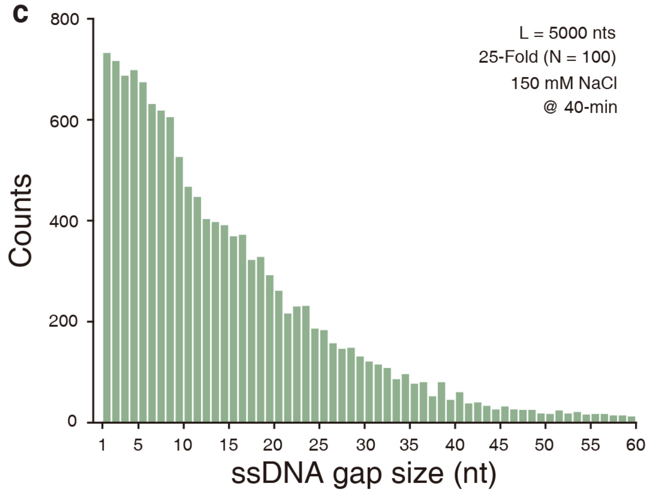

Supplementary Figure 6. Detailed analysis on RPA binding processes in the stochastic simulations. (**a**) Real-time changes of ssDNA region percentage bound by RPA in 20-nt mode (green), 30-nt mode (red) and no RPA (blue) with 0, 1, 4, 10, 25-Fold RPA at 150 mM NaCl (i) and 15 mM NaCl (ii) were plotted. (**b**) Quantification to the simulation results of the total number of bound RPA in 30-nt mode (# $RPA_{30\text{-}mode}$) and in 20-nt mode (# $RPA_{20\text{-}mode}$) on the ssDNA substrate, P_{20} values, and total size of naked ssDNA (nt) at 40-min time point, and loaded RPA number during 30-min to 40-min ($\Delta N_{RPA,30\text{-}40min}$) on 0, 1, 4, 10, 25-Fold RPA at 15 mM NaCl and 150 mM NaCl. (**c**) Distribution of ssDNA gap size (nt) on simulated 25-Fold RPA binding at 150 mM NaCl at 40-min time point. *N* represents the stochastic simulation for each condition was repeated for 100 times in a and c. Source data are provided as a Source Data file.

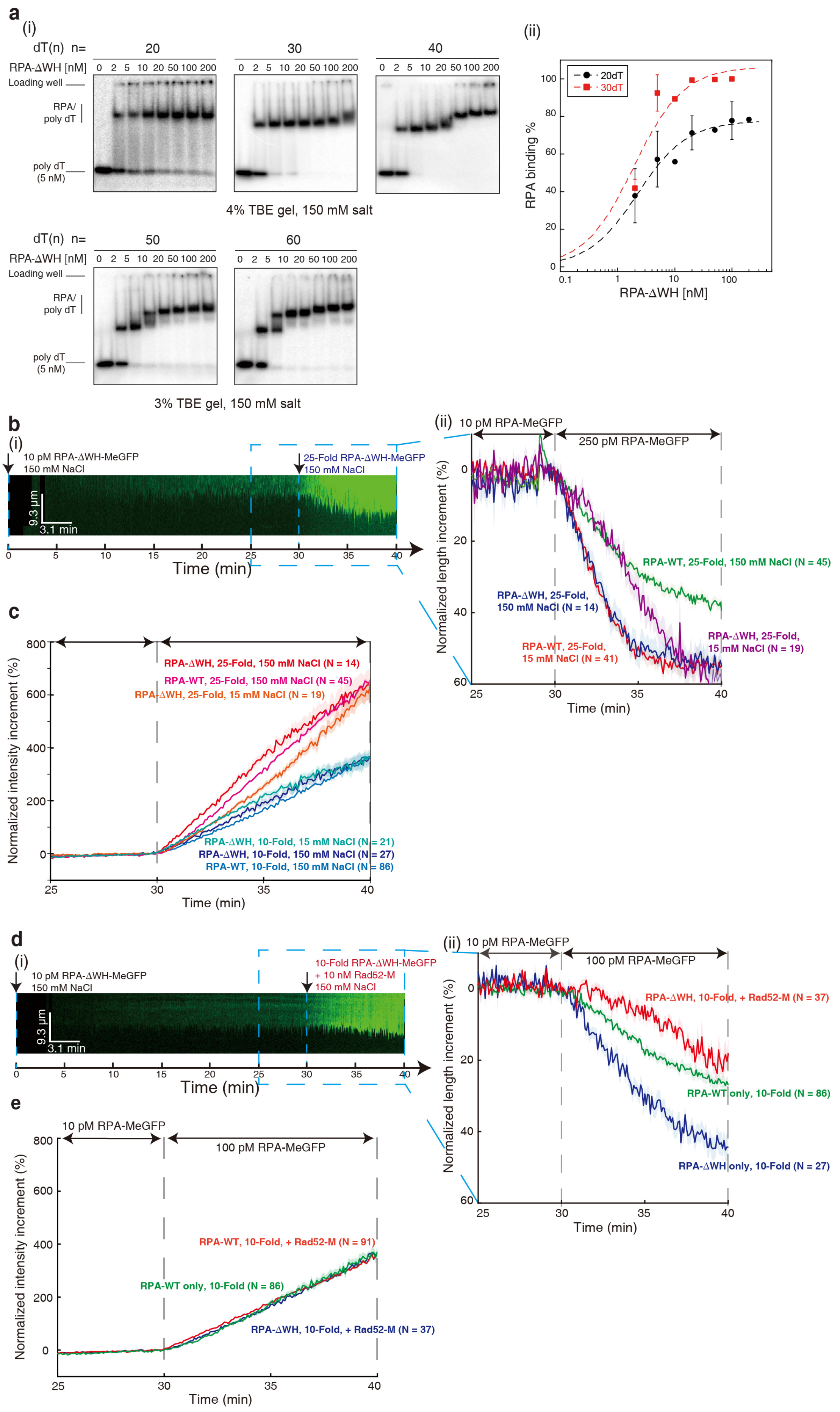
a
(i)
dT(n) n=
20
30
40
RPA-ΔWH [nM]
0 2 5 10 20 50 100 200
Loading well
RPA/ poly dT
poly dT (5 nM)
4% TBE gel, 150 mM salt
50
60
3% TBE gel, 150 mM salt
(ii)
20dT
30dT
RPA binding %
RPA-ΔWH [nM]
b
(i)
10 pM RPA-ΔWH-MeGFP 150 mM NaCl
25-Fold RPA-ΔWH-MeGFP 150 mM NaCl
9.3 μm
3.1 min
Time (min)
(ii) 10 pM RPA-MeGFP
250 pM RPA-MeGFP
Normalized length increment (%)
RPA-WT, 25-Fold, 150 mM NaCl (N = 45)
RPA-ΔWH, 25-Fold, 150 mM NaCl (N = 14)
RPA-WT, 25-Fold, 15 mM NaCl (N = 41)
RPA-ΔWH, 25-Fold, 15 mM NaCl (N = 19)
c
Normalized intensity increment (%)
RPA-ΔWH, 25-Fold, 150 mM NaCl (N = 14)
RPA-WT, 25-Fold, 150 mM NaCl (N = 45)
RPA-ΔWH, 25-Fold, 15 mM NaCl (N = 19)
RPA-ΔWH, 10-Fold, 15 mM NaCl (N = 21)
RPA-ΔWH, 10-Fold, 150 mM NaCl (N = 27)
RPA-WT, 10-Fold, 150 mM NaCl (N = 86)
Time (min)
d
(i)
10 pM RPA-ΔWH-MeGFP 150 mM NaCl
10-Fold RPA-ΔWH-MeGFP + 10 nM Rad52-M 150 mM NaCl
9.3 μm
3.1 min
Time (min)
(ii) 10 pM RPA-MeGFP
100 pM RPA-MeGFP
Normalized length increment (%)
RPA-ΔWH, 10-Fold, + Rad52-M (N = 37)
RPA-WT only, 10-Fold (N = 86)
RPA-ΔWH only, 10-Fold (N = 27)
Time (min)
e
10 pM RPA-MeGFP
100 pM RPA-MeGFP
Normalized intensity increment (%)
RPA-WT, 10-Fold, + Rad52-M (N = 91)
RPA-WT only, 10-Fold (N = 86)
RPA-ΔWH, 10-Fold, + Rad52-M (N = 37)
Time (min)

Supplementary Figure 7. RPA ssDNA binding mode is orchestrated through Rfa2 WH domain. (**a**) Titration of RPA-ΔWH (0-200 nM) to 5 nM 5’-labeled $dT_{(n)}$ ssDNA at 150 mM KCl, n = 20, 30, 40, 50, 60 (i), and quantification of RPA binding percent to $dT_{(20)}$ and $dT_{(30)}$ (ii). (**b**) Binding dynamics of RPA-ΔWH. Representative kymographs of 25-fold RPA-ΔWH at 150 mM NaCl were shown in (i), and its length analysis (blue) was in (ii) , together with 25-fold RPA-ΔWH at 15 mM NaCl (purple), where length dynamic curve with 25-fold RPA-WT at 150 mM NaCl (green) and 15 mM NaCl (red) condition from Fig. 3a-b were added for comparison. (**c**) Intensity analysis of RPA-ΔWH at 150 mM NaCl. 10-fold at 150 mM NaCl in red and at 15 mM NaCl in orange, and 25-fold at 150 mM NaCl in dark blue and at 15 mM NaCl in cyan. Intensity dynamic curve with 10-fold (pink) and 25-fold (light blue) RPA-WT at 150 mM NaCl from Supplementary Fig. 3a was added for comparison. (**d**) Effect of Rad52-M domain on RPA-ΔWH. Representative kymographs of 10-fold RPA-ΔWH/Rad52-M complex at 150 mM NaCl were shown in (i), and its length analysis (red) was in (ii), where length dynamic curve with 10-fold RPA-WT (green) from Fig. 3a or 10-fold RPA-ΔWH (blue) from Fig. 6b at 150 mM NaCl were added for comparison. RPA-ΔWH/Rad52-M complex was prepared by 30-min pre-incubation. (**e**) Intensity analysis of 10-fold RPA-WT/Rad52-M (red) and 10-fold RPA-ΔWH/Rad52-M (blue) at 150 mM NaCl. Intensity dynamic curve with 10-fold RPA-WT (green) at 150 mM NaCl from Supplementary Fig. 3a was added for comparison. *N* represents the total trace number of ssDNA-RPA complexes end tracking examined over three times DNA Curtains experiments for each experimental condition in b-e. *N* = 3 independent experiments for each experimental condition in a(ii). Data are presented as mean ± SD in a(ii), and mean ± SEM in b-e. Source data are provided as a Source Data file.

Supplementary References

1 Flory, P. J. Intramolecular reaction between neighboring substituents of vinyl polymers. *J Am Chem Soc* **61**, 1518-1521 (1939). https://doi.org:DOI 10.1021/ja01875a053

2 Mcghee, J. D. & Hippel, P. H. V. Theoretical Aspects of DNA-Protein Interactions - Cooperative and Non-Cooperative Binding of Large Ligands to a One-Dimensional Homogeneous Lattice. *J Mol Biol* **86**, 469-489 (1974). https://doi.org:Doi 10.1016/0022-2836(74)90031-X

3 Villaluenga, J. P. G., Vidal, J. & Cao-Garcia, F. J. Noncooperative thermodynamics and kinetic models of ligand binding to polymers: Connecting McGhee-von Hippel model with the Tonks gas model. *Phys Rev E* **102** (2020). https://doi.org:ARTN 012407, 10.1103/PhysRevE.102.012407

4 Jarillo, J. *et al.* Mechanics, thermodynamics, and kinetics of ligand binding to biopolymers. *Plos One* **12** (2017). https://doi.org:ARTN e0174830, 10.1371/journal.pone.0174830

5 Naufer, M. N. *et al.* Multiprotein E. coli SSB ssDNA complex shows both stable binding and rapid dissociation due to interprotein interactions. *Nucleic Acids Res* **49**, 1532-1549 (2021). https://doi.org:10.1093/nar/gkaa1267

6 Fanning, E., Klimovich, V. & Nager, A. R. A dynamic model for replication protein A (RPA) function in DNA processing pathways. *Nucleic Acids Res* **34**, 4126-4137 (2006). https://doi.org:10.1093/nar/gkl550

7 Caldwell, C. C. & Spies, M. Dynamic elements of replication protein A at the crossroads of DNA replication, recombination, and repair. *Crit Rev Biochem Mol* **55**, 482-507 (2020). https://doi.org:10.1080/10409238.2020.1813070

8 Bochkareva, E., Korolev, S., Lees-Miller, S. P. & Bochkarev, A. Structure of the RPA trimerization core and its role in the multistep DNA-binding mechanism of RPA. *Embo J* **21**, 1855-1863 (2002). https://doi.org:DOI 10.1093/emboj/21.7.1855

9 Fan, J. & Pavletich, N. P. Structure and conformational change of a replication protein A heterotrimer bound to ssDNA. *Genes & Development* **26**, 2337-2347 (2012). https://doi.org:10.1101/gad.194787.112

10 Bezanson, J., Edelman, A., Karpinski, S. & Shah, V. B. Julia: A Fresh Approach to Numerical Computing. *Siam Rev* **59**, 65-98 (2017). https://doi.org:10.1137/141000671

11 Gillespie, D. T. Exact Stochastic Simulation of Coupled Chemical-Reactions. *J Phys Chem-Us* **81**, 2340-2361 (1977). https://doi.org:DOI 10.1021/j100540a008
