# Supplementary material for: ssDNA accessibility of Rad51 is regulated by orchestrating multiple RPA dynamics
Source: Nat Commun. 2023 Jun 30;14:3864. doi: 10.1038/s41467-023-39579-y (PMC10313831; doi:10.1038/s41467-023-39579-y)
Supplement: Supplementary file 3 — Description of Additional Supplementary Files [file 41467_2023_39579_MOESM3_ESM.pdf]

Description of Additional Supplementary Files

File Name: Supplementary Data 1
Description: Value list of length and intensity analysis of all three-step ssDNA Curtains experiments conducted in this work.

File Name: Supplementary Movie 1
Description: Three-step low-complexity ssDNA Curtains. 10 pM RPA-MeGFP was flushed into the chamber for 30-min, and then 25-fold nM RPA-MeGFP (250 pM) was flushed into the chamber for another 10-min. The flow rate was 0.4 ml/min. The working buffer was 40 mM Tris-HCl (pH 7.5), 150 mM NaCl, 2 mM $MgCl_2$, and 0.2 mg/mL BSA.
