# Supplementary material for: ssDNA accessibility of Rad51 is regulated by orchestrating multiple RPA dynamics
Source: Nat Commun. 2023 Jun 30;14:3864. doi: 10.1038/s41467-023-39579-y (PMC10313831; doi:10.1038/s41467-023-39579-y)
Supplement: Supplementary file 6 — Reporting Summary [file 41467_2023_39579_MOESM6_ESM.pdf]

nature portfolio

Corresponding author(s): Hengyao Niu, and Zhi Qi

Last updated by author(s): May 24, 2023

Reporting Summary

Nature Portfolio wishes to improve the reproducibility of the work that we publish. This form provides structure for consistency and transparency in reporting. For further information on Nature Portfolio policies, see our Editorial Policies and the Editorial Policy Checklist.

Statistics

For all statistical analyses, confirm that the following items are present in the figure legend, table legend, main text, or Methods section.

n/a	Confirmed	
☐	☒	The exact sample size (*n*) for each experimental group/condition, given as a discrete number and unit of measurement
☐	☒	A statement on whether measurements were taken from distinct samples or whether the same sample was measured repeatedly
☐	☒	The statistical test(s) used AND whether they are one- or two-sided<br>*Only common tests should be described solely by name; describe more complex techniques in the Methods section.*
☒	☐	A description of all covariates tested
☐	☒	A description of any assumptions or corrections, such as tests of normality and adjustment for multiple comparisons
☐	☒	A full description of the statistical parameters including central tendency (e.g. means) or other basic estimates (e.g. regression coefficient) AND variation (e.g. standard deviation) or associated estimates of uncertainty (e.g. confidence intervals)
☐	☒	For null hypothesis testing, the test statistic (e.g. *F*, *t*, *r*) with confidence intervals, effect sizes, degrees of freedom and *P* value noted<br>*Give P values as exact values whenever suitable.*
☒	☐	For Bayesian analysis, information on the choice of priors and Markov chain Monte Carlo settings
☒	☐	For hierarchical and complex designs, identification of the appropriate level for tests and full reporting of outcomes
☒	☐	Estimates of effect sizes (e.g. Cohen's *d*, Pearson's *r*), indicating how they were calculated

Our web collection on statistics for biologists contains articles on many of the points above.

Software and code

Policy information about availability of computer code

Data collection	All experimental data of DNA Curtains were acquired with a custom-built prism-type total internal reflection fluorescence microscope (TIRFM) (Nikon, Inverted Microscope Eclipse Ti-E). The software was CellVision Coolight Technology Version 1.4.0 (Home-made software).
Data analysis	1. Batch analysis of images was performed using Open source image processing software ImageJ (1.52P)(http://imagej.net/Contributors), MATLAB 2021a software (https://www.mathworks.com/products/matlab.html), and Python (3.7.0)(https://www.python.org).<br>2. The stochastic simulation model for multiple RPA molecules binding to long ssDNA was conducted by custom code in Julia (1.8.0) (https://github.com/hsianktin/RPA_model).

For manuscripts utilizing custom algorithms or software that are central to the research but not yet described in published literature, software must be made available to editors and reviewers. We strongly encourage code deposition in a community repository (e.g. GitHub). See the Nature Portfolio guidelines for submitting code & software for further information.

Data

Policy information about availability of data

All manuscripts must include a data availability statement. This statement should provide the following information, where applicable:

- Accession codes, unique identifiers, or web links for publicly available datasets
- A description of any restrictions on data availability
- For clinical datasets or third party data, please ensure that the statement adheres to our policy

The data that support the findings of this study are available from the corresponding author upon reasonable request. The source data underlying Figs 1-7, Supplementary Figs 1-7 are provided as a Source Data file.

Human research participants

Policy information about studies involving human research participants and Sex and Gender in Research.

Reporting on sex and gender	Not relevant, because our work was an in vitro biophysical and biochemical study.
Population characteristics	Not relevant, because our work was an in vitro biophysical and biochemical study.
Recruitment	Not relevant, because our work was an in vitro biophysical and biochemical study.
Ethics oversight	Not relevant, because our work was an in vitro biophysical and biochemical study.

Note that full information on the approval of the study protocol must also be provided in the manuscript.

Field-specific reporting

Please select the one below that is the best fit for your research. If you are not sure, read the appropriate sections before making your selection.

☒ Life sciences ☐ Behavioural & social sciences ☐ Ecological, evolutionary & environmental sciences

For a reference copy of the document with all sections, see nature.com/documents/nr-reporting-summary-flat.pdf

Life sciences study design

All studies must disclose on these points even when the disclosure is negative.

Sample size	No statistical method was performed to predetermine sample sizes. For the high-throughput ssDNA Curtains experiments, the sample size was determined by the molecules acquired while assuring adequately sampling the behavior of molecules. Our results won't change if increase the sample size, which indicated our sample size was sufficient. All experiments were repeated at least three times. Figures show one representative experiment. All the stochastic simulations were repeatedly sampled for 100 times.
Data exclusions	No data were excluded in this study.
Replication	This was an in vitro biophysical study, and all replication attempts were successful. All biochemical experiments including SDS-PAGE, EMSAs, were repeated three times. All the ssDNA Curtains experiments were repeated three times. All stochastic simulations were repeated 100 times.
Randomization	Not relevant, because our work was an in vitro biophysical and biochemical study. Our experimental subjects were molecules rather than human beings. They can be split into aliquots soon after they were purified or synthesized to make sure the samples for each experiments were at the same state.
Blinding	Not relevant, because our work was an in vitro biophysical and biochemical study. Our experimental subjects were molecules rather than human beings, therefore, no blinding is needed for the experimental design.

Reporting for specific materials, systems and methods

We require information from authors about some types of materials, experimental systems and methods used in many studies. Here, indicate whether each material, system or method listed is relevant to your study. If you are not sure if a list item applies to your research, read the appropriate section before selecting a response.

Materials & experimental systems

n/a	Involved in the study
☒	☐ Antibodies
☒	☐ Eukaryotic cell lines
☒	☐ Palaeontology and archaeology
☒	☐ Animals and other organisms
☒	☐ Clinical data
☒	☐ Dual use research of concern

Methods

n/a	Involved in the study
☒	☐ ChIP-seq
☒	☐ Flow cytometry
☒	☐ MRI-based neuroimaging
